# Immune memory in convalescent patients with asymptomatic or mild COVID-19

**DOI:** 10.1038/s41421-021-00250-9

**Published:** 2021-03-25

**Authors:** Quan-Xin Long, Yan-Jun Jia, Xin Wang, Hai-Jun Deng, Xiao-Xia Cao, Jun Yuan, Liang Fang, Xu-Rong Cheng, Chao Luo, An-Ran He, Xiao-Jun Tang, Jie-li Hu, Yuan Hu, Ni Tang, Xue-Fei Cai, De-Qiang Wang, Jie Hu, Jing-Fu Qiu, Bei-Zhong Liu, Juan Chen, Ai-long Huang

**Affiliations:** 1 Key Laboratory of Molecular Biology on Infectious Diseases, Ministry of Education, Chongqing Medical University, Chongqing, 400016 China; 2 Department of Endocrinology and Metabolism, The Second Affiliated Hospital of Chongqing Medical University, Chongqing, 400010 China; 3 Yongchuan Hospital Affiliated to Chongqing Medical University, Chongqing, 402160 China; 4 Wanzhou District Center for Disease Control and Prevention, Chongqing, 404100 China; 5 School of Public Health and Management, Chongqing Medical University, Chongqing, 400016 China

**Keywords:** Immunology, Mechanisms of disease

## Abstract

It is important to evaluate the durability of the protective immune response elicited by primary infection with severe acute respiratory syndrome coronavirus 2 (SARS-CoV-2). Here, we systematically evaluated the SARS-CoV-2-specific memory B cell and T cell responses in healthy controls and individuals recovered from asymptomatic or symptomatic infection approximately 6 months prior. Comparatively low frequencies of memory B cells specific for the receptor-binding domain (RBD) of spike glycoprotein (S) persisted in the peripheral blood of individuals who recovered from infection (median 0.62%, interquartile range 0.48-0.69). The SARS-CoV-2 RBD-specific memory B cell response was detected in 2 of 13 individuals who recovered from asymptomatic infection and 10 of 20 individuals who recovered from symptomatic infection. T cell responses induced by S, membrane (M), and nucleocapsid (N) peptide libraries from SARS-CoV-2 were observed in individuals recovered from coronavirus disease 2019 (COVID-19), and cross-reactive T cell responses to SARS-CoV-2 were also detected in healthy controls.

## Introduction

Coronavirus disease 2019 (COVID-19), caused by severe acute respiratory syndrome coronavirus 2 (SARS-CoV-2) infection^1^, is a global pandemic, with more than 102 million infections and more than 2,209,000 deaths as of 1 Feb 2021, according to the COVID-19 report of the World Health Organization. The clinical manifestations of SARS-CoV-2 infection range from asymptomatic disease or mild symptoms to severe pneumonia, acute respiratory distress syndrome (ARDS), and even death^2^. Adaptive immunity^3^, including humoral and cellular immune responses, has been proven to be a crucial step in viral infection control. In SARS-CoV-2 infection, adaptive immune responses^4–7^ also present a prominent role in infection eradication, which is similar to the situation for other respiratory viral infections.

Although SARS-CoV-2-specific antibodies and neutralizing antibodies develop rapidly after infection^8,9^, recent studies suggest that antibodies mounted against SARS-CoV-2 do not persist over time and decline several weeks following the onset of symptoms^10–13^. Human memory B cell response is supposed to be long-lived, but different viral infections lead to variations in the duration of memory cells. Specific memory B cell responses to variola virus, varicella-zoster, measles, and mumps were estimated to persist over 50 years^14^. Other viral infections, such as influenza^15^ and respiratory syncytial virus (RSV)^16^, confer a waning immunological memory response. For recovered SARS-CoV infection patients followed up for 6 years, there was no peripheral memory B cell response^17^. To date, there is scattered evidence of reinfection by SARS-CoV-2^18,19^, and reinfections by natural infection occur for all four seasonal coronaviruses^20^. It is unclear whether a potential anamnestic B cell response exists and whether this response is strong enough to protect a person from reinfection after rechallenge with SARS-CoV-2.

Across current studies, the basic observation in patients with COVID-19 is robust T cell activation and cycling responding at a variably high frequency to epitopes across the majority of the viral proteome. Responding T cells show an activation phenotype, including the expression of Ki67, CD38, and human leukocyte antigen-DR (HLA-DR)^6,21,22^, while elevated expression of exhaustion markers, including programmed cell death protein (PD-1) and T cell immunoglobulin mucin-3 (TIM-3), has also been reported in some studies^23,24^. Peripheral SARS-CoV-2-specific memory CD4^+^ T cells and CD8^+^ T cells were detected in 100 and 70% of convalescent individuals following the above 3-week infection, respectively^25^. Memory T cell responses were detected in patients who recovered from SARS followed up 1, 2 and even 4 years later, with a clear decline over time^26,27^. The durability of protective T cell memory following either SARS-CoV-2 infection or vaccination is a key question.

The objectives of the current study were to describe the dynamics of antibodies, measure the antigen-specific B cell compartment, and evaluate the memory B cell and T cell response in patients recovered from SARS-CoV-2 infection 6 months after infection. This study will provide insights into the protective capacity of immune memory, including humoral and cellular memory, that contributes to the duration of protection against reinfection after natural infection and the durability of vaccine protection.

## Results

### Subject characteristics

Recovered COVID-19 subjects were adults with a prior positive COVID-19 PCR test and met the definition of recovery based on the guideline from the Chinese Center for Disease Control and Prevention. Healthy donors were adults with no prior diagnosis or recent symptoms consistent with COVID-19 and with negative SARS-CoV-2 serological test results. Twenty individuals recovered from COVID-19 with symptoms (RS), 13 individuals recovered from asymptomatic infection (RA), and 10 healthy controls (HC) were recruited in this study (Supplemental Table S1). The clinical parameters of these patients did not show differences compared with healthy donors (Supplemental Table S2). The median follow-up days of symptomatic recovered individuals and asymptomatic recovered individuals were 169 (interquartile range (IQR): 168–174) days and 170 (IQR: 164–174) days, respectively. Six months after infection, SARS-CoV-2-specific IgG turned negative in 3 of the 13 individuals who recovered from asymptomatic infection, while 2 of the 20 individuals who recovered from symptomatic infection showed seronegative characteristics, as measured by the current chemiluminescent immunoassay (CLIA) (23.1% vs 10%, *P* = 0.360). The virus-specific antibody levels and neutralizing antibody levels showed no difference at 6 months after infection between the asymptomatic and symptomatic recovered groups (*P* = 0.074 and *P* = 0.870, respectively) (Supplementary Fig. S1a, b). The white blood cell count, neutrophil count, and monocyte count were obviously lower in SARS-CoV-2-infected recovered individuals than in the healthy controls (Supplementary Fig. S2a–c).

### Memory B cell repertoire in individuals recovered from SARS-CoV-2

**Fig. 1:**
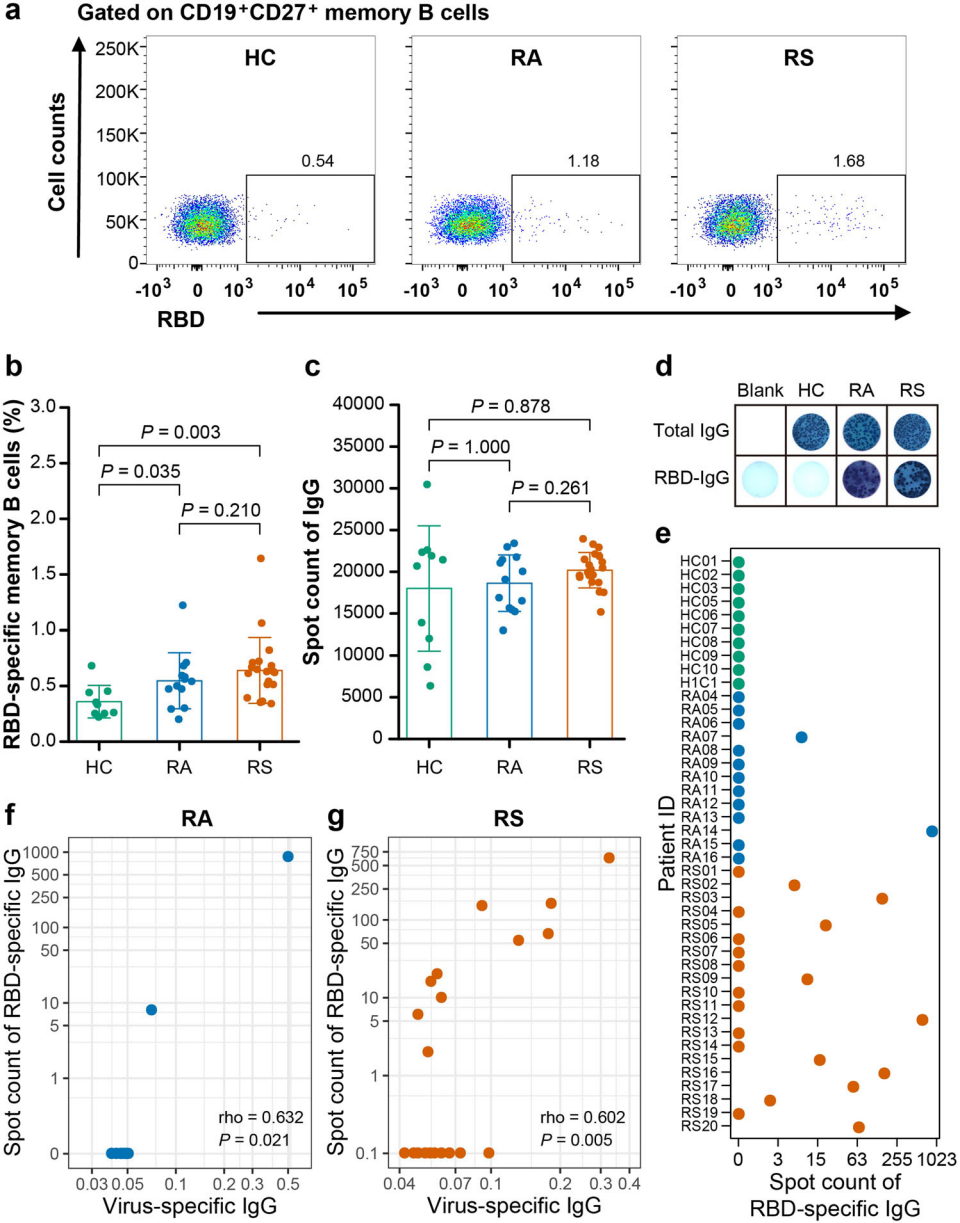
Memory B cell repertoire in individuals recovered from COVID-19. **a**, **b** Flow cytometry analysis (**a**) of the percentages (**b**) of SARS-CoV-2 RBD-specific memory B cells within the CD19^+^ CD27^+^ memory B cells of the PBMCs from recovered individuals and healthy controls (HC). Freshly isolated PBMCs were stimulated in vitro for 5 days with R848 and IL-2. After 5 days in culture, these cells were plated onto commercial ELISpot plates. **c** The counts of cells that produced total IgG in per million cultured PBMCs from recovered individuals and healthy controls. **d** Representative ELISpot dots in recovered individuals and healthy controls. **e** The counts of cells that produced RBD-specific IgG in per million cultured PBMCs from recovered individuals and healthy controls. **f** Correlation analysis between RBD-specific ELISpot dot number and virus-specific IgG levels in supernatant after the R848 stimulation on PBMC from individuals recovered from asymptomatic. **g** Correlation analysis between RBD-specific ELISpot dot number and virus-specific IgG levels in supernatant after the R848 stimulation on PBMC from individuals recovered from symptomatic. Each symbol represents an individual throughout. Data in **b**, **c** were analyzed using unpaired, two-sided Mann–Whitney U test, and data in **f**, **g** were analyzed using Spearman’s rank correlation test.

Memory B cells are of great importance for long-term humoral immunity. To define the memory B cell response to SARS-CoV-2, peripheral blood mononuclear cells (PBMCs) from recovered patients and healthy controls were isolated and analyzed by flow cytometry. There was no difference in the percentages of naïve B cells (CD19^+^CD21^+^CD27^-^), total memory B cells (CD19^+^CD27^+^), activated memory B cells (CD19^+^CD21^-^CD27^+^CD38^-/low^), and antibody-secreting B cells (CD19^+^CD21^-^CD27^+^CD38^+/high^) between the recovered individuals and control subjects (Supplementary Fig. S3a–d). The frequencies of SARS-CoV-2 virus-specific memory B cells were further evaluated by fluorescent receptor-binding domain (RBD) antigen (Fig. 1a). As shown in Fig. 1b, the proportion of virus-specific memory B cells in individuals recovered from symptoms and in asymptomatic patients was obviously higher than that in healthy controls (0.62 vs 0.33, *P* = 0.003; 0.54 vs 0.33, *P* = 0.035). However, there was no significant difference in the proportion of virus-specific memory B cells between individuals who recovered from symptoms and asymptomatic individuals (0.62 vs 0.54, *P* = 0.210) (Fig. 1b).

In addition, to define the functional properties of virus-specific memory B cells, memory B cells in whole PBMC cultures were selectively activated and expanded with IL-2 and R848 as stimulation^28^, and the numbers of memory B cells that produce IgG and are able to bind the virus RBD were counted with a commercial ELISpot kit. As shown in Fig. 1c, the non-specific B cell responses, presented as the total spots of B cells producing IgG, were not significantly different in the three groups (RA vs HC, *P* = 1.000; RS vs HC, *P* = 0.878) (Fig. 1c). The SARS-CoV-2-specific B cell responses were presented as RBD-specific IgG. As shown in Fig. 1d, e, 2 individuals (15.4%, 2 of 13) recovered from asymptomatic infection were positive for RBD-specific IgG production, and the number of spots per million PBMCs was 8 and 866, while 10 individuals (50%, 10 of 20) recovered from symptomatic infection were positive for producing RBD-specific IgG, and the number of spots ranged from 2 to 616 per million PBMCs (median 37, IQR 12–129) (Fig. 1e). There was no RBD-specific blot in healthy controls (Fig. 1e). The SARS-CoV-2 IgG levels in the supernatant after stimulation with IL-2 and R848 were also qualified by Enzyme-linked immunosorbent assay (ELISA), and a correlation between the SARS-CoV-2-specific blot numbers and virus-specific IgG levels in the supernatant was found (RA, Spearman’s rank correlation *ρ* = 0.632, *P* = 0.021; RS, Spearman’s rank correlation *ρ* = 0.602, *P* = 0.005) (Fig. 1f, g). There was no correlation between spot number after memory B cell activation and circulating SARS-CoV-2-specific IgG levels or virus-specific memory B cells in peripheral blood from convalescent individuals (Supplementary Fig. S4a, b).

### T cell subsets in COVID-19 convalescent individuals

**Fig. 2:**
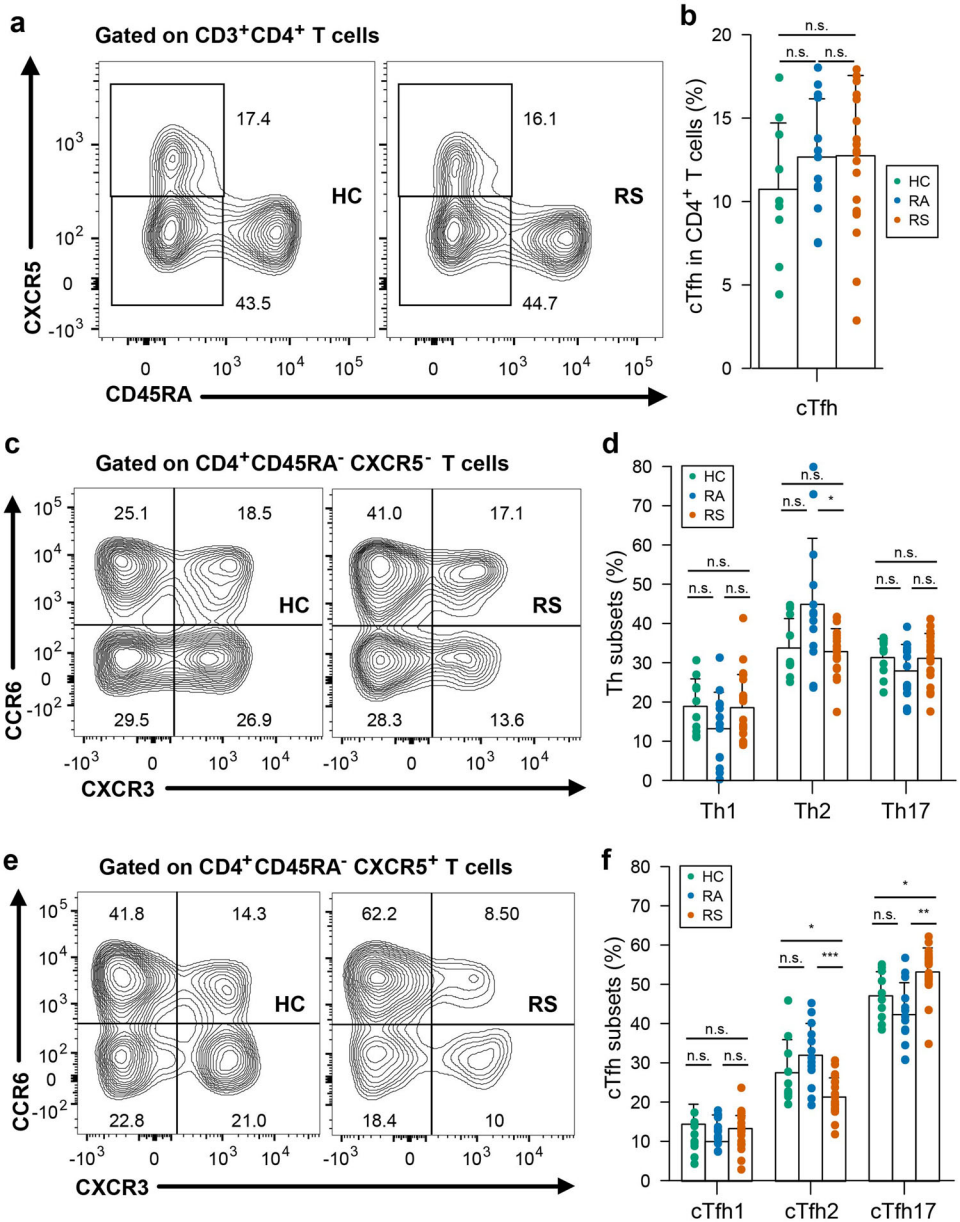
T cell subsets in individuals recovered from COVID-19. Blood samples were collected from individuals recovered from COVID-19 and healthy controls, PBMC were isolated for analysis of T cell subsets. **a** Representative flow plots for the expression of CD45RA and CXCR5 within the CD4^+^ populations from healthy controls and recovered COVID-19. **b** Proportions of cTfh cells (CD45RA^-^CXCR5^+^) in CD4^+^ T cell populations from HC, RS and RA. **c** Representative flow plots for the expression of CXCR3 and CCR6 within the CD4^+^CD45RA^-^CXCR5^-^populations from healthy controls and recovered COVID-19. **d** Proportions of Th subsets within the CD4^+^CD45RA^-^CXCR5^-^ populations from HC, RS, and RA. **e** Representative flow plots for the expression of CXCR3 and CCR6 within the cTfh populations from healthy controls and recovered COVID-19. **f** Proportions of cTfh subsets within the cTfh populations from HC, RS, and RA. Each symbol represents an individual throughout. RS, individuals recovered from COVID-19 with symptoms (*n* = 20); RA, individuals recovered from asymptomatic (*n* = 13); HC, healthy controls (*n* = 10). Data in **b**, **d**, **f** were analyzed using unpaired, two-sided Mann–Whitney U test. n.s., not significant.

Patients infected with SARS-CoV-2 exhibit disrupted T cell homeostasis in the acute phase. To explore the cellular immune responses to SARS-CoV-2, we first evaluated T cell subsets in the peripheral blood isolated from COVID-19 convalescent subjects and controls. There was no significant difference in the frequencies of total CD3^+^, CD4^+^, or CD8^+^ T cells within the T cell population between convalescent patients and controls (Supplementary Fig. S5a–c). In addition, in either the CD4^+^ or CD8^+^ T pools from convalescent subjects, the distribution of naïve (CD45RA^+^CCR7^+^), central memory (CD45RA^-^CCR7^+^), and effector memory (CD45RA^-^CCR7^-^) T cells was similar to that in the T cell pools from healthy controls (Supplementary Fig. S5d–i). Circulating follicular helper T (cTfh) cells facilitate the antibody response to viral infections^29^, and cTfh cells in the circulation constitute a surrogate of cTfh cells in lymphoid tissues^30^. A recent study showed that convalescent patients display an altered peripheral CD4^+^ T cell spectrum, especially for cTfh cells^31^. As shown in Fig. 2a, b, we found that there is no difference in the frequency of cTfh between convalescent patients and healthy controls. We also found that despite the similar proportions of circulating Th1, Th2, and Th17 cells within CD4^+^ T cells between convalescent patients and healthy controls (Fig. 2c, d), cTfh2 frequency in individuals recovered from symptoms was lower than those in healthy controls (with median 20.1% vs 23.8%, *P* = 0.028), while cTfh17 population abundance was higher than those in healthy controls (with median 54.1% vs 47.0%, *P* = 0.016) (Fig. 2e, f).

### Identification and quantitation of the SARS-CoV-2-specific T cell response in individuals recovered from COVID-19

**Fig. 3:**
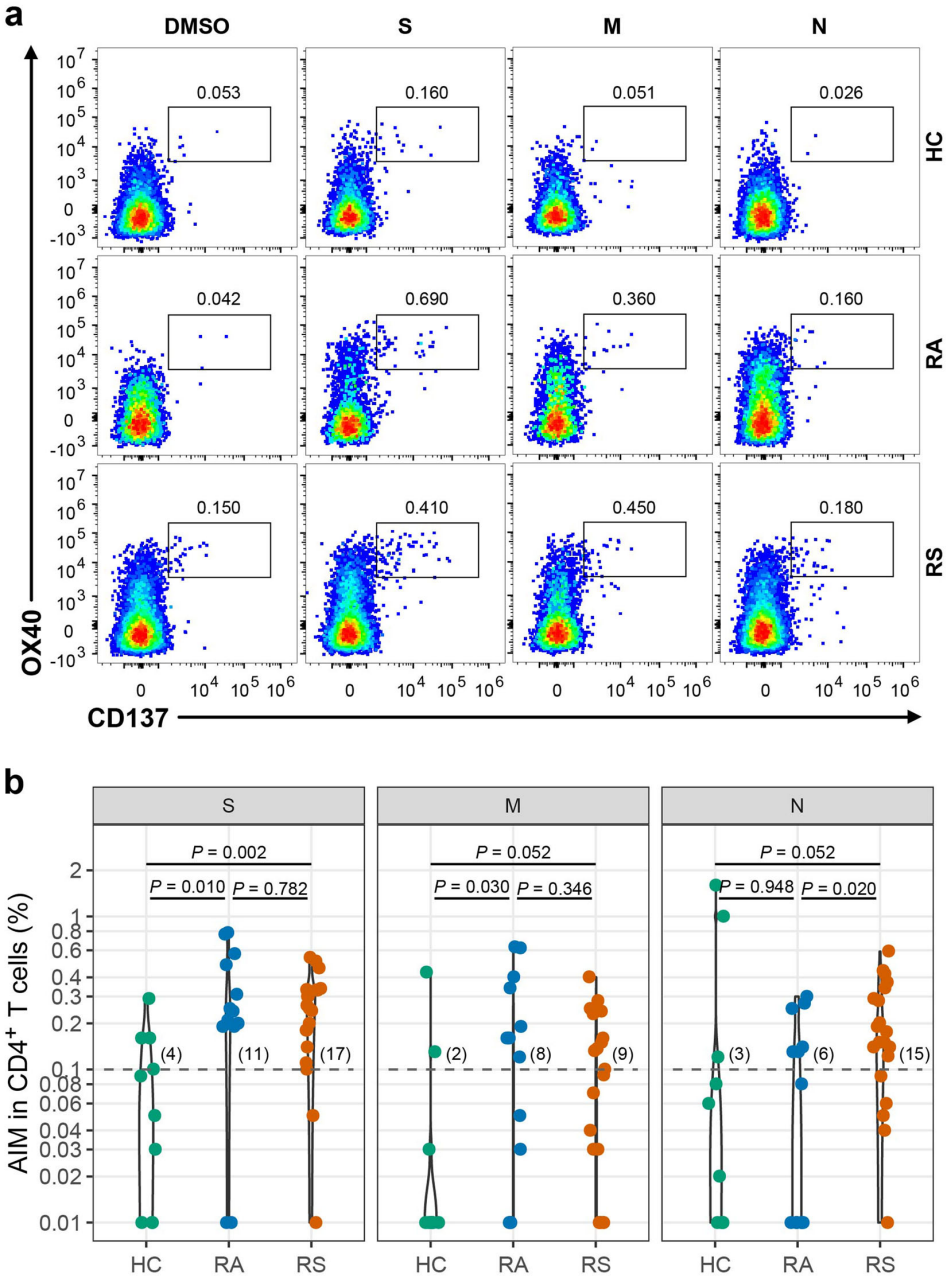
SARS-CoV-2 specific CD4^+^ T cell response of recovered COVID-19 individuals. **a** Representative flow plots for the expressions of OX40 and CD137 within CD3^+^CD4^+^ T cells of cultured PBMCs stimulated with SARS-CoV-2 peptide library in different subjects. **b** SARS-CoV-2-specific CD4^+^ T cells measured as percentage of AIM^+^ (OX40^+^CD137^+^) after stimulation of PBMCs with peptide pools encompassing S, M, and N. Each symbol represents an individual throughout. RS, individuals recovered from COVID-19 with symptoms (*n* = 20); RA, individuals recovered from asymptomatic (*n* = 13); HC, healthy controls (*n* = 10). Data were background subtracted against DMSO negative control, and if the value is greater than 0.1%, the individual is considered as response to peptide library stimulation. The number of response individuals is shown in brackets, and the dotted line indicated the cut-off for positive responder. The comparisons between two groups were performed by using unpaired, two-sided Mann–Whitney U test. S, spike; M, membrane; N, nucleocapsid.

To delineate the memory response mediated by T cells, PBMCs from recovered individuals and healthy controls were stimulated with a spike (S), membrane (M), and nucleocapsid (N) peptide libraries from SARS-CoV-2 ex vivo. Phytohemagglutinin-L (PHA-L) was used as a positive control, while DMSO was used as the negative control. As previously reported, the TCR-dependent activation-induced marker (AIM) was used to identify and quantify virus-specific CD4^+^ (co-expression of OX40^+^CD137^+^) or CD8^+^ T (co-expression of CD69^+^CD137^+^) cells in recovered COVID-19 individuals^25^. As shown in Fig. 3a, b, a SARS-CoV-2 S-specific CD4^+^ T cell response (OX40^+^CD137^+^) was detected in 17 of 20 individuals recovered from COVID-19 with symptoms (fisher’s exact *P* = 0.030 vs healthy controls, 4 of 10 individuals), and a CD4^+^ T cell response to M and N was also detected in individuals recovered from symptoms, with 9 (45%) and 15 (75%) individuals recovered from COVID-19 with symptoms, respectively (Fig. 3b), which were not significantly different than healthy controls (fisher’s exact *P* were 0.246 and 0.045 vs healthy controls, respectively). The magnitudes of the SARS-CoV-2-specific CD4^+^ T cell responses measured were 0.26 (IQR: 0.17–0.33), 0.10 (IQR: 0.02–0.18), and 0.16 (IQR: 0.11–0.30) after stimulation with S, M, and N pool, respectively (Fig. 3b). In individuals recovered from COVID-19 without symptoms, CD4^+^ T cell responses to S, M and N were detected in 11, 8 and 6 of 13 individuals (Fig. 3b) (fisher’s exact *P* were 0.039, 0.090 and 0.669 vs healthy controls) with magnitudes of 0.24 (IQR: 0.19–0.48), 0.16 (IQR: 0.03–0.34) and 0.08 (IQR: 0.01–0.14), respectively (Fig. 3b). The CD4^+^ T cell response to N measured by AIM was higher in individuals recovered from symptoms than in individuals recovered from asymptomatic symptoms (*P* = 0.020), whereas the CD4^+^ T cell responses to S and M were not different (*P* = 0.782 and 0.346 for S and M, respectively) between subjects recovered from symptomatic infection and recovered from asymptomatic infection (Fig. 3b). The rate of subjects with CD4^+^ T cell responses simultaneously to S, M, and N was 0% (0/10), 30.8% (4/13), and 45% (9/20) in HC, RA, and RS, respectively, and individuals recovered from SARS-CoV-2 infection have obviously higher rate of CD4^+^ T cell response compared with HC.

**Fig. 4:**
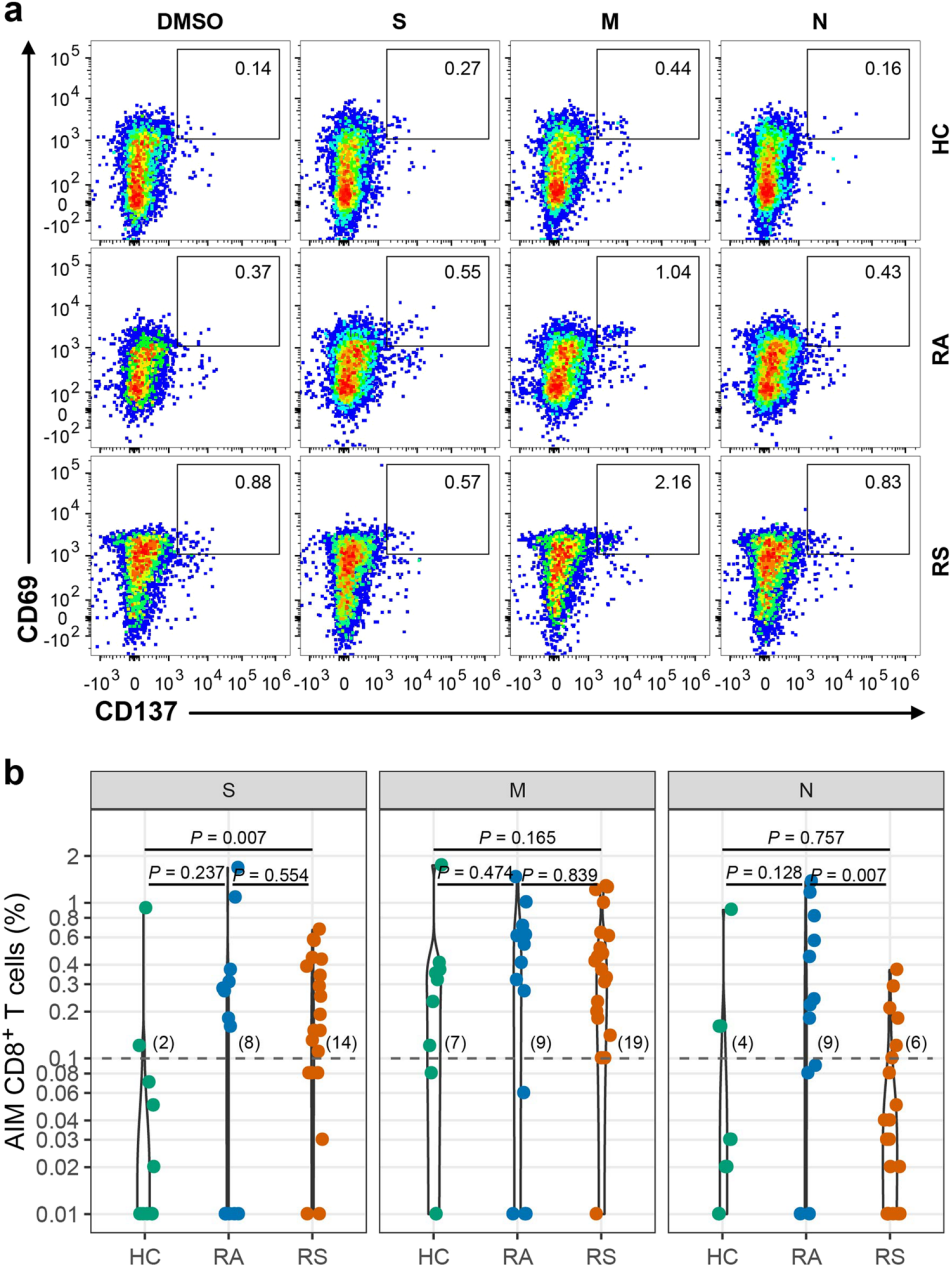
SARS-CoV-2-specific CD8^+^ T cell response of recovered COVID-19 individuals. **a** Representative flow plots for the expressions of CD69 and CD137 within CD3^+^CD4^+^ T cells of cultured PBMCs stimulated with SARS-CoV-2 peptide library in different subjects. **b** SARS-CoV-2-specific CD8^+^ T cells measured as percentage of AIM^+^ (CD69^+^CD137^+^) after stimulation of PBMCs with peptide pools encompassing S, M, and N. Each symbol represents an individual throughout. RS, individuals recovered from COVID-19 with symptoms (*n* = 20); RA, individuals recovered from asymptomatic (*n* = 13); HC, healthy controls (*n* = 10). Data were background subtracted against DMSO negative control, and if the value is greater than 0.1%, the individual is considered as a response to peptide library stimulation. The number of response individuals is shown in brackets, and the dotted line indicated the cut-off for positive responder. The comparisons between two groups were performed by using unpaired, two-sided Mann–Whitney U test. S, spike; M, membrane; N, nucleocapsid.

SARS-CoV-2 S-, M-, and N-specific CD8^+^ T cell responses (CD69^+^CD137^+^) were detected in 14, 19, and 6 individuals recovered from COVID-19 with symptoms, and the magnitudes of the SARS-CoV-2-specific CD8^+^ T cell responses measured were 0.17 (IQR: 0.08–0.40), 0.40 (IQR: 0.19–0.62) and 0.04 (IQR: 0.01–0.11), respectively (Fig. 4a, b). In individuals recovered from COVID-19 without symptoms, CD8^+^ T cell responses to S, M, and N were detected in 8, 9, and 9 of 13 individuals (Fig. 4b) (fisher’s exact *P* were 0.090, 1.000, and 0.222 vs healthy controls), with magnitudes of 0.18 (IQR: 0.01–0.31), 0.41 (IQR: 0.06–0.62) and 0.24 (IQR: 0.09–0.82), respectively (Fig. 4b). The CD8^+^ T cell response to N was higher in individuals recovered from asymptomatic infection than in individuals recovered from symptomatic infection (*P* = 0.007), and the CD8^+^ T cell responses to S and M were not different between them (*P* = 0.554 and 0.839 for S and M, respectively) (Fig. 4b). The rate of subjects with CD8^+^ T cell responses simultaneously to S, M, and N was 10% (1/10), 38.5% (5/13), and 20% (4/20) in HC, RA, and RS, respectively, and individuals recovered from SARS-CoV-2 infection have obviously higher rate of CD8 + T cell response compared with HC.

**Fig. 5:**
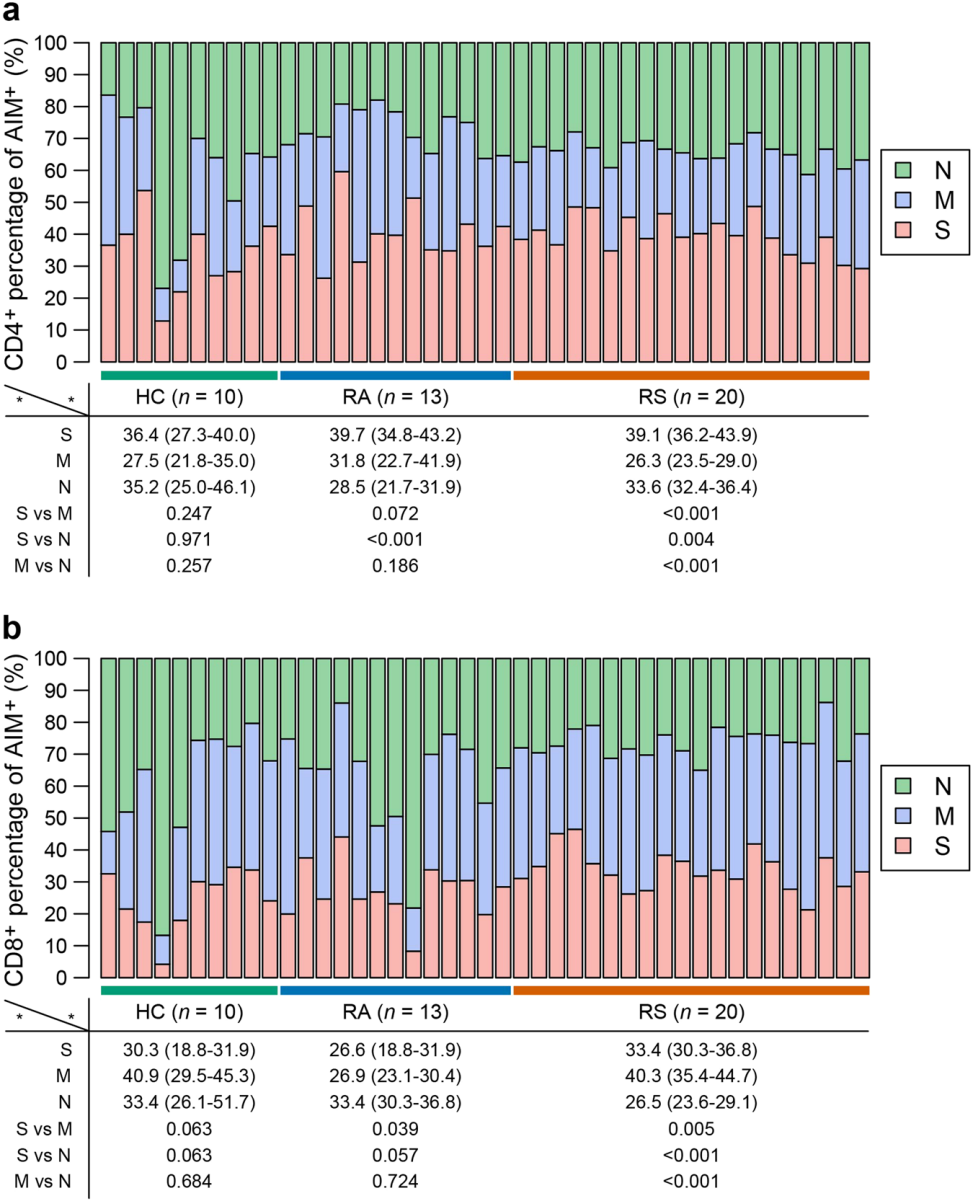
The composition of SARS-CoV-2 response in each individual is shown as a percentage of the total detected T cell response. **a** The composition of SARS-CoV-2-specific CD4^+^ T cell response (OX40^+^CD137^+^) in individuals. **b** The composition of SARS-CoV-2-specific CD8^+^ T cell response (CD69^+^CD137^+^) in individuals. The percentage of S, M, and N-specific CD4^+^ or CD8^+^ T cell response was shown as median (IQR), and were performed by using two-sided Mann–Whitney U test. S, spike; M, membrane; N, nucleocapsid. RS, individuals recovered from COVID-19 with symptoms (*n* = 20); RA, individuals recovered from asymptomatic (*n* = 13); HC, healthy controls (*n* = 10).

Regarding the distribution of the T cell response for different epitopes evaluated from the AIM assay, nearly 40% of the CD4^+^ T cell response was against the S peptide library, while the M peptide library stimulated the strongest CD8^+^ T cell response in individuals who recovered from symptoms, suggesting that the distribution of epitopes was different in different SARS-CoV-2 proteins (Fig. 5a, b). Either CD4^+^ or CD8^+^ T cell subsets might preferentially respond to different peptide libraries.

**Fig. 6:**
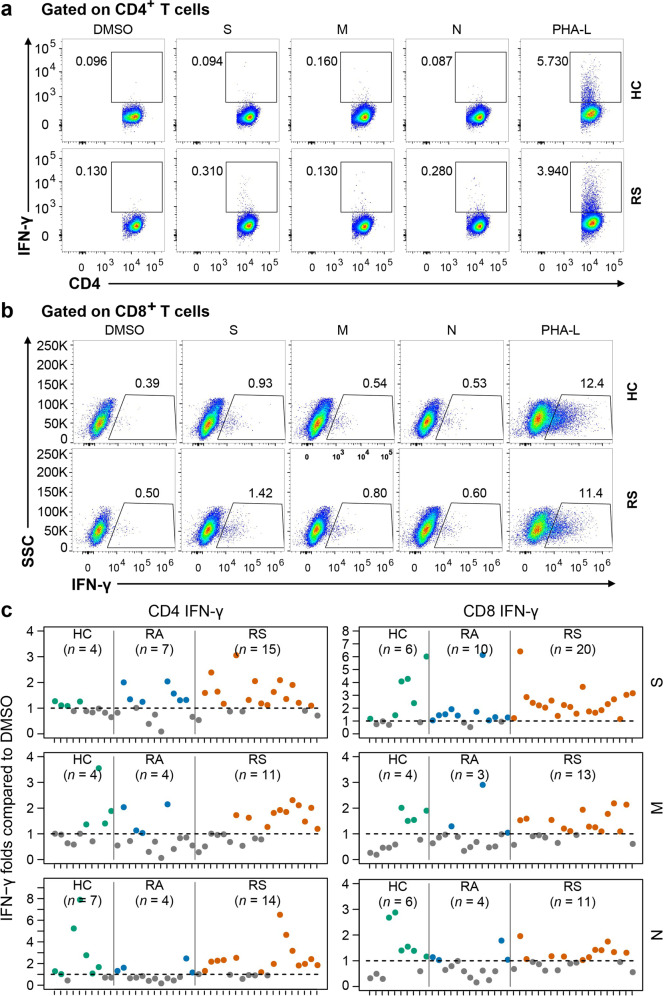
Percentage of IFN-γ-producing T cell in response to different peptide library. Isolated PBMCs were stimulated with specific peptide library of SARS-CoV-2 for 24 h, and the proportions of IFN-γ-producing T cell within the CD4^+^ or CD8^+^ T cell population were determined with intracellular staining. **a**, **b** Representative flow plots for the expressions of IFN-γ within CD3^+^CD4^+^ T cells (**a**) or CD3^+^CD8^+^ T cells (**b**) of cultured PBMCs from healthy controls and recovered COVID-19. **c** Fold changes between percentage of IFN-γ^+^ producing CD4^+^ or CD8^+^ T cells stimulated with SARS-CoV-2 peptide library and with DMSO. Number of stimulation response individuals (fold change > 1) are depicted in brackets. The dotted line indicated the cut-off for positive responder. Each symbol represents an individual throughout. S, spike; M, membrane; N, nucleocapsid. RS, individuals recovered from COVID-19 with symptoms (*n* = 20); RA, individuals recovered from asymptomatic (*n* = 13); HC, healthy controls (*n* = 10).

Independently, to further confirm the functional repertoire of the SARS-CoV-2-specific T cell response, PBMCs from recovered individuals and healthy controls were directly stimulated with different peptide pools for 24 h, and the percentage of T cells producing interferon-γ (IFN-γ) was determined with intracellular staining. As shown in Fig. 6, all individuals who recovered from symptoms had a clear population of CD8^+^ T cells that produced IFN-γ, while CD4^+^ T cells producing IFN-γ were detectable in 15 of 20 individuals stimulated with S peptide library. Thus, the majority of recovered COVID-19 patients generated a specific T cell response against S protein of SARS-CoV-2 after rechallenge.

### T cell ELISpot

**Fig. 7:**
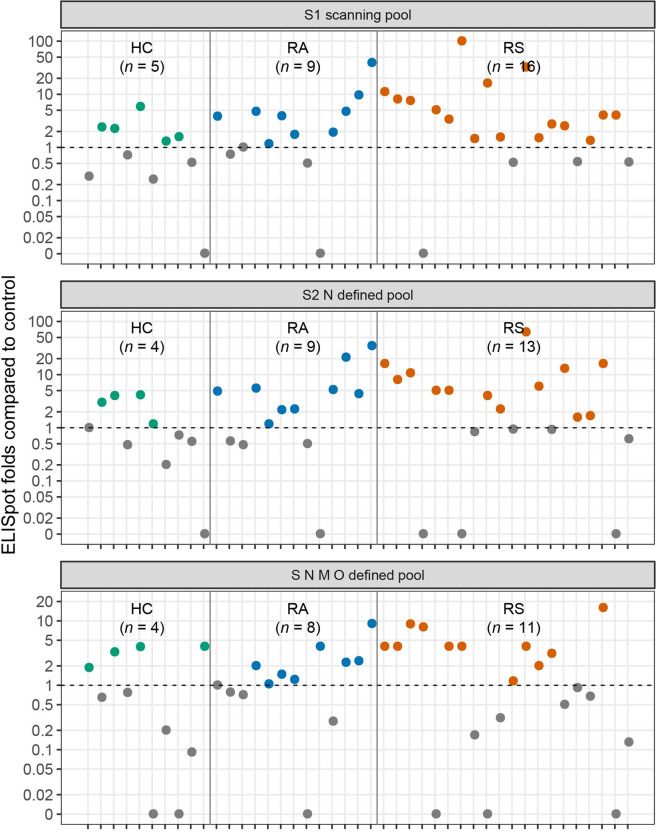
The SARS-CoV-2-specific T cell response measured by ELISpot in COVID-19 recovered individuals. ELISpot results shown as the fold changes of peptide stimulation were presented as the spot number ratio between peptide stimulation and DMSO stimulation. Number of stimulation response individuals (fold change > 1) are depicted in brackets. The dotted line indicated the cut-off for positive responder. RS, individuals recovered from COVID-19 with symptoms (*n* = 20); RA, individuals recovered from asymptomatic (*n* = 13); HC, healthy controls (*n* = 10).

To further explore the virus-specific memory T cell response, PBMCs from recovered individuals and healthy controls were stimulated with different peptide pools (Mabtech, S1 scanning pool, S2 N defined pool and S N M O defined pool, peptide constitutions were listed in Supplementary Table S3), and virus-specific responses were analyzed by commercial IFN-γ ELISpot assay. In individuals recovered from COVID-19 with symptoms, 16, 13, and 11 out of 20 individuals showed reactivity against the S1, S2 N, and S N M O peptide pools, while in individuals recovered from COVID-19 without symptoms, 9, 9, and 8 out of 13 individuals showed reactivity against the S1, S2 N, and S N M O peptide pools. The fold changes of peptide stimulation are presented as the spot-forming unit ratio between DMSO stimulation and peptide stimulation (Fig. 7). The S1 pool had higher reactivity in COVID-19 recovered individuals than in healthy controls (S1 scanning pool: RS vs HC, *P* = 0.028; RA vs HC, *P* = 0.154).

## Discussion

The induction and duration of SARS-CoV-2-specific memory T cells and B cells are important for long-term protection and are also key issues in addressing reinfection episodes and the duration of protection induced by vaccines. There is a critical need to evaluate the capacity of adaptive immune memory in individuals recovered from COVID-19. Here, we utilized PBMCs derived from recovered COVID-19 patients to determine the memory response mediated by B cells and T cells by a series of experimental techniques, including phenotype analysis, functional measurement with ELISpot, intracellular cytokine staining and T cell AIM assessment. Consistent with recent studies, a cross-reactivity in memory reaction for coronavirus mediated by T cells was observed in healthy controls^7,25,32^.

To the best of our knowledge, this is the first study to evaluate the memory B cell response to SARS-CoV-2 in individuals infected 6 months prior. Although virus-specific memory B cells in different cohorts were all at low levels, similar to a previous study^33^, the level of SARS-CoV-2-specific memory B cells in SARS-CoV-2-infected individuals was obviously higher than those in healthy controls. After stimulation with the combination of R848 and IL-2^28^, memory B cells were selectively activated, and RBD-specific IgG was further detected as the ELISpot readout. In our study, we found that only 2 of 13 individuals who recovered from asymptomatic infection presented positive results, while 6 of 20 individuals who recovered from symptomatic infection presented positive results, suggesting that not every infected person can produce an effective humoral immune response to the virus. This result is extremely important for vaccine development and application. The magnitude of B cell spot number was not correlated with the levels of virus-specific IgG in peripheral blood. These results indicated that memory B cell activation, differentiation, or antibody-secreting B cell (i.e., plasmablasts and plasma cells) formation may be deficient or out of balance in COVID-19-recovered individuals. cTfh cells indicated maturation of the humoral immune response and were related to the establishment of specific memory B cells to rapidly respond to possible reinfection. In the present cohort, we found that despite cTfh frequency was similar between recovered subjects symptoms and healthy control, convalescent subjects displayed skewed cTfh subsets differentiation, with increased cTfh17 while decreased cTfh2 population. Interestingly, Jennifer A. Juno et al. recently reported that recovered patients (one month after infection) exhibited robust cTfh responses to SARS-CoV-2, and virus-specific cTfh cells were enriched for cTfh17 subsets^34^. Thus, we speculate that abnormal distribution of cTfh pools might be involved in the pathogenesis or protective reaction of COVID-19. However, the extremely low number of virus-specific circulating cTfh cells in convalescent subjects, especially in this study with recovered individuals 6 months after infection, largely impedes from further investigating the roles of specific cTfh subsets in virus immunity.

SARS-CoV-2-specific T cell responses were detected in the majority of individuals recovered from SARS-CoV-2 infection 6 months prior, similar to recent preprint^35,36^. In previous studies that investigated individuals recovered from SARS-CoV infection, memory T cells were shown to persist for many years^7,17^. In our study, AIM, ICS, and T cell activation marker screening was conducted using large peptide mega pools to evaluate the T cell response in individuals infected 6 months prior to avoid the potential differences induced by different methods. In the CD4^+^ T cell response, S protein accounted for nearly 40% of the total CD4^+^ T cell response in individuals recovered from symptomatic infection, which was obviously higher than the CD4^+^ T cell response induced by M or N (39.1% vs 26.3%, *P* < 0.001; 39.1% vs 33.6%, *P* = 0.004). Although the CD8^+^ T cell response was observed in both recovered cohorts with different peptide library stimulations, the positive rate was not significantly higher than that in healthy controls. This discrepancy in the T cell response in SARS-CoV-2 infection convalescents may support additional focus on eliciting the CD4^+^ T cell response using exogenous antigens in vaccine development, as is the case in herpes zoster vaccines^37^.

The difference of humoral and cellular immune response in subjects recovered from different disease severity was also a hotspot. In virus-specific memory B cell functional test, the rate of individuals with positive B cell ELISpot result were higher in RS group compared with RA group (50.0% vs 15.4%) (Fig. 1f, g). Memory CD4^+^ or CD8^+^ T cell frequencies stimulated with S or M peptide library showed no difference (Figs. 3b, 4b). Therefore, the long-term humoral immunity to SARS-CoV-2 was higher in individuals who experienced a severe COVID-19 disease course, while T cell memory did not show a similar pattern^38^. However, a recent preprint by Zuo J. et al. reported that symptomatic infection elicits a higher level of memory T cells than asymptomatic infection at 6-month post-infection^35^, this discrepancy might be due to the heterogeneity in the study population^39^.

Importantly, cross-reactive T cell responses against S, M, or N peptide library were detected in 40.0% (4 of 10 individuals), 20.0% (2 of 10 individuals), and 30.0% (3 of 10 individuals) of healthy controls, which is consistent with previous studies reporting pre-existing immune responses potentially induced by other coronaviruses^7,25,32,40^. In the H1N1 pandemic, the presence of cross-reactive T cells was found to correlate with less severe disease^41,42^, but a preprint study reported that pre-existing humoral immunity to common coronaviruses does not confer cross-protection against SARS-CoV-2 infection^43^. The possible effects of pre-existing T cells on the susceptibility to SARS-CoV-2, differential modulation of SARS-CoV-2 infection severity, epidemiological models of herd immunity, and the performance of COVID-19 candidate vaccines need to be carefully evaluated, especially when SARS-CoV-2-specific T cell epitope is available^44^.

Specific memory B cell populations have the ability to re-enter secondary germinal centers (GCs) to play roles upon recall immunization^45^. Therefore, virus-specific memory B cells residing in other parts of the body, such as the bone marrow and secondary lymphoid organs, need to be inspected if possible. In addition, in the current study, the longitudinal analysis of the dynamic kinetics of memory B cell and T cell responses was hampered by the lack of stored PBMC samples during acute infection. Furthermore, our study did not recruit individuals who recovered from severe conditions because severe COVID-19 patients were scarce in local medical institutions. The lack of antigen-specific tetramers makes this study unable to directly analyze the proportion of antigen-specific memory T cell subsets. Finally, the relatively small cohort size is also a limitation of the current study.

## Methods and subjects

### Study subjects

Subjects recovered from asymptomatic (*n* = 13) or symptomatic COVID-19 (*n* = 20) at least 6 months were recruited in Wanzhou District (13/33) and Yongchuan District (20/33), Chongqing, China (Supplementary Table S1). Ten healthy controls were recruited to provide EDTA-K_2_ anticoagulant blood samples. All plasma was obtained by centrifuging blood samples at 3500 rpm for 5 min and frozen at –80 °C for further analysis. The study was approved by the Ethics Committee of Chongqing Medical University. Written informed consent in accordance with the Declaration of Helsinki from all participants.

### Cell isolation

PBMCs were isolated from EDTA-K_2_ anticoagulant whole blood using Ficoll-Hypaque (GE Healthcare, USA) gradient centrifugation.

### T cells stimulation

2 × 10^6^ freshly isolated PBMCs were cultured in 48-well plate (Corning) in RPMI 1640 medium supplemented with 10% (wt/vol) fetal bovine serum (FBS), 1 mM sodium pyruvate, 10 mM HEPES buffer solution, 100 μM nonessential amino acid solution, 50 μM β-mercaptoethanol, 100 U/mL penicillin and 100 µg/mL streptomycin and stimulated with 1 μg/mL functional grade anti-CD28 (eBioscience, clone 28.2) and 1 μg/mL SARS-CoV-2 Spike Glycoprotein, SARS-CoV-2 M or SARS-CoV-2 N (Genscript, China) at 37 °C, 5% CO_2_ for 24 h. Stimulation controls were both conducted in the presence of 1 µg/mL anti-CD28 and separately conducted with equal concentrations of DMSO (Sigma) as vehicle control, or 2.5 μg/mL PHA-L Solution (eBioscience) with as positive controls.

### B cells stimulation

2 × 10^6^ PBMCs were cultured in 12-well plate (Corning) at 37 °C, 5% CO_2_ in the presence of 1 μg/mL R848 (Mabtech) and 10 ng/mL IL-2 (Mabtech) as previously described. After incubation for 5 days, cells were collected for analysis of spot numbers of producing IgG specific for SARS-CoV-2 RBD, and cell supernatant was used to determine IgG levels.

### Flow cytometry

For analysis of surface marker, fresh PBMCs were incubated for 30 min at 4 °C in PBS containing 2% FBS with the fluorochrome conjugated antibodies titrated to optimal concentrations. Fixable Viability Dye eFluor 780 (eBioscience) staining was used to exclude dead cells. The SARS-CoV-2-specific B cells were detected using biotinylated S1-His recombinant protein (SinoBiological) and Streptavidin-APC (Biolegend). For intracellular cytokine (IFN-γ) staining, surface stained cells were fixed and permeabilized with a Cytofix/Cytoperm kit (BD Biosciences) according to the manufacturer’s instruction. All samples were acquired on BD FACSAria^TM^ II (BD Biosciences) and analyzed using FlowJo software (Version 10.0.8, Tree Star Inc., USA). All antibodies used in this study were listed in Supplementary Table S4. The specific gating strategies for each population are indicated in each figure legend.

### T cell ELISpot

IFN-γ-secreting T cells were detected by Human IFN-γ SARS-CoV-2 ELISpotPLUS (HRP) kit (Mabtech) according to the manufacture protocol. Briefly, 2.5 × 10^5^ cells were incubated with 1 μg/mL SARS-CoV-2 S1 scanning peptides, SARS-CoV-2 S N M O defined peptides or SARS-CoV-2 S2 N defined peptides (Mabtech) supplemented with 1 μg/mL anti-CD28 (eBioscience, clone 28.2) for 24 h. Unstimulated controls were performed with equal concentrations of DMSO, and positive control was incubated with 1 μg/mL CD3 (5 × 10^4^ cells, Mabtech, clone CD3-2) in the presence of 1 µg/mL anti-CD28. After 24 h treatment, spots were counted using an ELISpot Reader system (AID). Spots numbers were converted into the number of spots per million cells.

### B cell ELISpot

Numbers of B cells secreting IgG (total IgG) antibodies or IgG specific for the SARS-CoV-2 RBD were conducted with Human IgG SARS-CoV-2 RBD ELISpotPLUS (HRP) kit (Mabtech) according to the manufacture protocol. Spots numbers were converted into the number of spots per million cells.

### ELISA

After stimulation mentioned above, the levels of IgG specific for SARS-CoV-2 Spike S1 recombinant protein in cell supernatant were determined by ELISA according to the manufacturer’s instructions (SinoBiological).

### Detection of IgG against SARS-CoV-2

All serum samples were inactivated at 56 °C for 30 min and stored at –80 °C before testing. SARS-CoV-2 specific IgG against SARS-CoV-2 in plasma samples was tested using magnetic chemiluminescence enzyme immunoassay kits supplied by Bioscience Co. (approved by the China National Medical Products Administration; approval numbers 20203400183(IgG)), according to the manufacturer’s instructions. Briefly, recombinant antigens containing the nucleoprotein and a peptide (LQPELDSFKEELDKYFKNHTSPDVD) from the spike protein of SARS-CoV-2 were immobilized on magnetic particles. Antibody levels are presented as the measured chemiluminescence values divided by the cutoff (S/CO).

### Neutralization detection

Neutralization detection using pseudovirus neutralizaion assay was carried out as previously described^10^. Briefly, a codon-optimized S protein that lacked the C-terminal 19 amino acids was used to generate a luciferase-expressing pseudovirus, and the SARS-CoV-2 pseudovirus neutralization assay was carried out on 293 T cells expressing ACE2 in a 96-well plate. All serum was diluted at 1:160 and neutralization rate was calculated in the previous work^10^.

### Clinical parameters

All clinical parameters listed in Supplementary Table S2 were determined by professionals in the clinical laboratory followed Standard Operating Procedure.

### Statistical analysis

Continuous variables are presented as the median (IQR), and the comparison between two groups was evaluated using the two-tailed, non-parametric Mann-Whitney U test; categorical variables are presented as numbers (%), and the comparison between two groups was assessed using χ² test or Fisher’s exact test. For the correlation analyses, Spearman’s rank correlation was performed. The box plots show the medians (middle line) and the first and third quartiles (boxes), and the bar plots show the means ± SD. *P* value less than 0.05 was considered statistically significant. All statistical analyses were performed using R software, version 3.6.0.

## Supplementary information

Supplementary Information
