# Supplementary material for: Immune memory in convalescent patients with asymptomatic or mild COVID-19
Source: Cell Discov. 2021 Mar 25;7:18. doi: 10.1038/s41421-021-00250-9 (PMC7993859; doi:10.1038/s41421-021-00250-9)
Supplement: Supplementary file 1 — Supplementary Information [file 41421_2021_250_MOESM1_ESM.pdf]

Supplemental information

Supplementary Figure S1

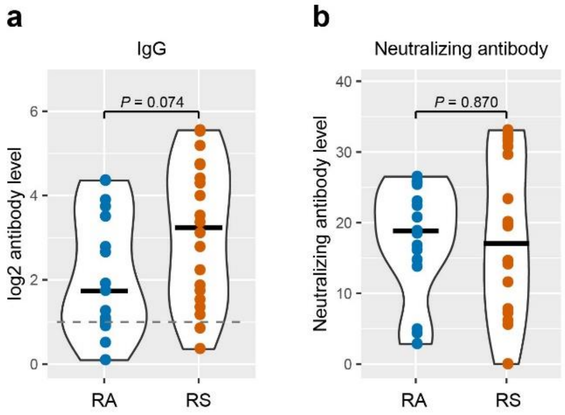

Supplementary Figure S2

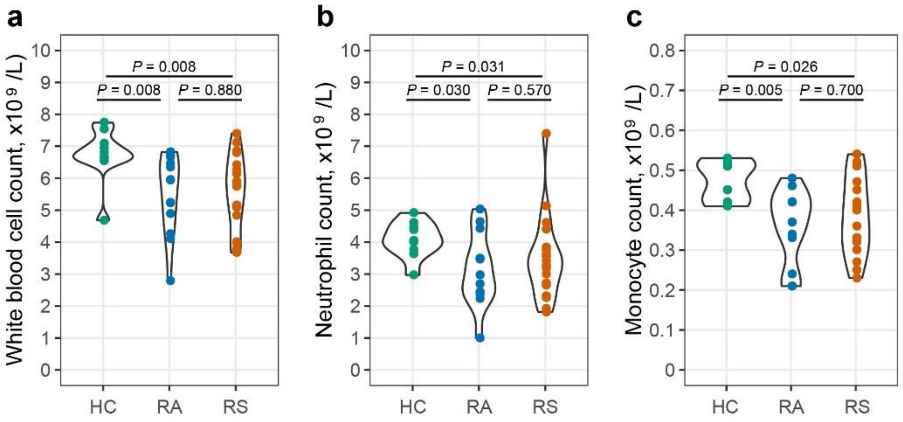

Supplementary Figure S3

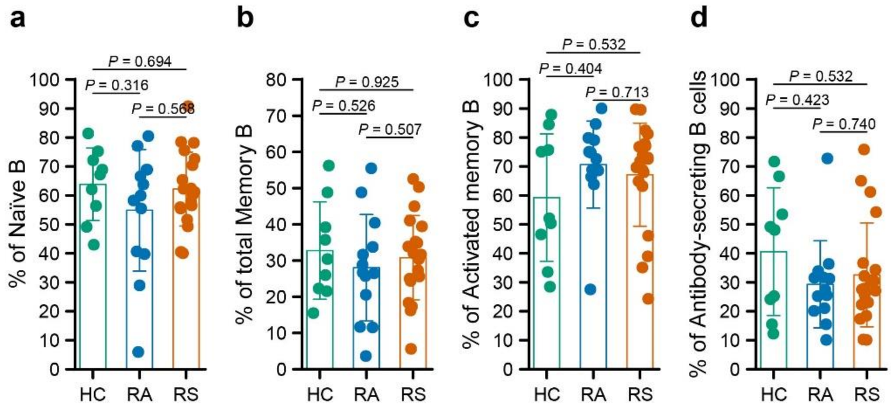

Supplementary Figure S4

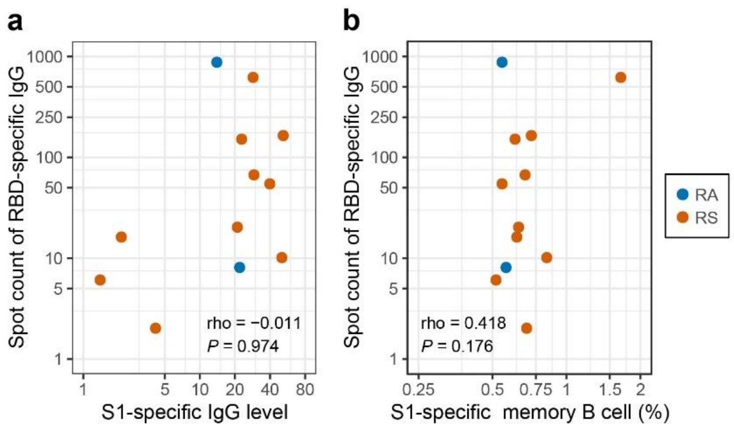

Supplementary Figure S5

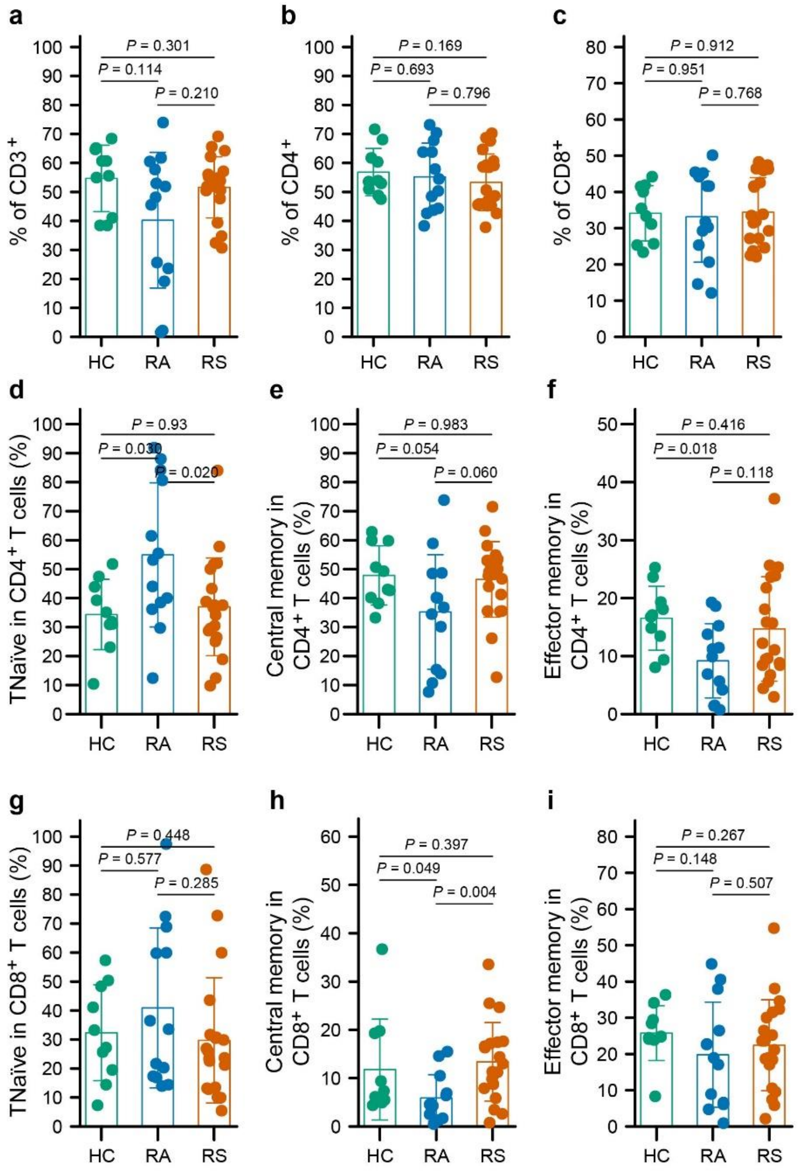

Supplementary Figure legends

Supplementary Fig S1. SARS-CoV-2 IgG and neutralization antibody level in individuals recovered from COVID-19 with/without symptoms. a Plasma SARS-CoV-2 specific IgG levels in individuals recovered from COVID-19. Data are shown as log2 (S/CO+1). **b** Plasma neutralization antibody level in individuals recovered from COVID-19. Data are shown as inhibition rate. Samples were from individuals recovered from symptomatic COVID-19 (RS, *n* = 20), individuals recovered from asymptomatic COVID-19 (RA, *n* = 13). Each symbol represents an individual throughout. Data were analyzed using unpaired, two-sided Mann-Whitney U test.

Supplementary Fig S2. Blood Cell counts in healthy controls and individuals recovered from COVID-19. a White blood cell count in different subjects. **b** Neutrophil count in different subjects. **c** Monocyte count in different subjects. Each symbol represents an individual throughout. Data was analyzed using unpaired, two-sided Mann-Whitney U test.

Supplementary Fig S3. B cell subsets in subjects recovered from SARS-CoV-2. a Percentage of naïve B cells within $CD19^+$ B cell of PBMCs from different subjects. **b** Percentage of memory B cells ($CD27^+$) within $CD19^+$ B cell of PBMCs different subjects. **c, d** Percentage of activated memory B cell ($CD21^-CD27^+CD38^{-/low}$) (**c**) and (**d**) Percentage of antibody secreting B cell ($CD21^-CD27^+CD38^{+/high}$) within $CD19^+CD21^-CD27^+$ B cell of PBMCs different subjects. Each symbol represents an individual

throughout. Data was analyzed using unpaired, two-sided Mann-Whitney U test, presented as mean ± SD.

Supplementary Fig S4. The correlation analysis between RBD specific ELISpot dot number and virus specific IgG level/virus specific memory B cell number. a The correlation analysis between RBD specific ELISpot dot number and virus specific IgG level in individuals recovered from symptomatic. **b** The correlation analysis between RBD specific ELISPOT dot number and percentage of virus specific memory B cells in individuals recovered from symptomatic. The correlation was analyzed using Spearman's rank correlation test. Samples with countable ELISpot dot number were taken into this analysis (RA, n = 2, RS, n = 10).

Supplementary Fig S5. Peripheral T cell subsets in COVID-19 convalescent individuals. a The frequencies of CD3$^+$ T cells in PBMCs from different individuals. **b, c** The frequencies of CD4$^+$ T cells (**b**) and CD8$^+$ T cells (**c**) within CD3$^+$ T cell pool in PBMCs from different individuals. **d-f** Proportions of naive T cell (CD45RA$^+$CCR7$^+$) (**d**), central memory T cell (CD45RA$^-$ CCR7$^+$) (**e**), and effector memory T cell (CD45RA−CCR7−) subsets (**f**) in CD4$^+$ T cell populations from healthy controls and recovered COVID-19. **g-i** Proportions of naive T cell (**g**), central memory T cell (**h**), and effector memory T cell subsets (**i**) in CD8$^+$ T cell populations from healthy controls and recovered COVID-19. Each symbol represents an individual throughout. Data was analyzed using unpaired, two-sided Mann-Whitney U test.

Supplemental Table S1. Clinical character statistics enrolled of this study.

Characteristics	RS (*n* = 20)	RA (*n* = 13)	HC (*n* = 10)
**Age (median, IQR)**	49.5 (44.8-55.8)	47.0 (27.0-55.0)	38.5 (27.0-52.8)
**Sex (Male/Female)**			
Male	9 (45.0%)	4 (30.8%)	6 (60.0%)
Female	11 (55.0%)	9 (69.2%)	4 (40.0%)
**Exposure**			
From Wuhan	2 (10.0%)	1 (7.7%)	-
Close contacts	18 (90.0%)	12 (92.3%)	-
**Comorbidities**			
Hypertension	4 (20.0%)	2 (15.4%)	-
Cardiovascular disease	2 (10.0%)	-	-
Diabetes	3 (15.0%)	1 (7.7%)	-
Chronic liver disease	2 (10.0%)	1 (7.7%)	-
Any	9 (45.0%)	3 (23.1%)	-
**Signs and symptoms**			
Fever	4 (20.0%)	-	-
Fatigue	1 (5.0%)	-	-
Dry cough	7 (35.0%)	-	-
Inappetence	2 (10.0%)	-	-
Dyspnea	2 (10.0%)	-	-
Expectoration	2 (10.0%)	-	-
Pharyngalgia	2 (10.0%)	-	-
Diarrhea	1 (5.0%)	-	-
Dizziness	1 (5.0%)	-	-
Vomiting	1 (5.0%)	-	-
Chills	2 (10.0%)	-	-
Rhinorrhea	2 (10.0%)	-	-
Chest stuffiness	1 (5.0%)	-	-

RS: Recovered from symptomatic infection, RA: Recovered from asymptomatic infection, HC: Healthy controls.

Supplemental Table S2. Laboratory findings of individuals in this study.

Variables	RS (*n* = 20)	RA (*n* = 13)	HC (*n* = 10)	RS vs. HC	RA vs. HC	RS vs. RA
**Blood routine examination**						
Red blood cell count, × 10^{12}/L	4.61 (4.15-5.01)	4.37 (4.27-4.79)	4.84 (4.57-4.93)	0.663	0.313	0.715
White blood cell count, × 10^9/L	5.75 (4.96-6.34)	5.94 (4.89-6.45)	6.73 (6.59-7.05)	0.008	0.008	0.880
Neutrophils count, × 10^9/L	3.28 (2.67-3.80)	2.97 (2.42-3.49)	4.03 (3.83-4.45)	0.031	0.030	0.570
Lymphocytes count, × 10^9/L	1.62 (1.44-1.94)	1.86 (1.38-2.47)	1.99 (1.74-2.33)	0.098	0.828	0.478
Monocyte cell count, × 10^9/L	0.40 (0.32-0.46)	0.37 (0.33-0.42)	0.48 (0.43-0.51)	0.026	0.005	0.700
Platelets count, × 10^9/L	162.00 (139.00-213.00)	198.00 (157.00-213.00)	229.00 (208.25-275.50)	0.008	0.088	0.318
Haemoglobin, g/L	135.00 (121.50-146.00)	129.00 (124.00-138.00)	143.50 (135.50-147.50)	0.550	0.402	1.000
**Blood biochemistry**						
Urea nitrogen, nmol/L	5.60 (4.86-7.00)	4.83 (4.41-5.79)	5.51 (5.29-6.11)	0.965	0.284	0.217
Creatinine, μmol/L	72.20 (66.70-83.12)	64.10 (58.80-67.40)	68.55 (65.52-82.90)	0.775	0.186	0.068
Glucose, mmol/L	4.88 (3.83-7.22)	3.69 (3.06-5.17)	4.38 (4.13-6.30)	0.880	0.154	0.128
Uric acid, μmol/L	299.20 (250.02-331.05)	276.70 (224.50-382.20)	314.05 (296.73-376.22)	0.328	0.232	0.730
Total protein, g/L	83.30 (81.35-86.15)	78.20 (77.50-80.40)	81.10 (77.45-85.90)	0.333	0.306	0.008
Albumin, g/L	44.00 (42.40-45.23)	45.40 (42.10-45.80)	45.85 (45.17-47.08)	0.099	0.291	0.417
Globulin, g/L	39.90 (37.10-42.08)	33.00 (31.80-35.90)	35.05 (33.22-39.00)	0.118	0.402	0.002
Alanine aminotransferase, U/L	23.50 (18.00-33.75)	15.00 (8.00-19.00)	19.50 (10.75-26.25)	0.271	0.264	0.010
Aspartate aminotransferase, U/L	33.00 (26.75-41.50)	22.00 (20.00-31.00)	46.50 (35.00-49.75)	0.094	0.002	0.007
γ-glutamyl transpeptidase, U/L	23.00 (11.75-41.00)	16.00 (14.00-17.00)	30.50 (22.25-36.75)	0.226	0.005	0.386

Total bilirubin, μmol/L	7.95 (5.85-11.82)	7.20 (6.70-8.70)	7.95 (7.60-9.70)	0.843	0.402	0.407
Direct bilirubin, μmol/L	4.40 (3.27-5.48)	3.00 (2.20-3.60)	3.85 (3.37-4.27)	0.390	0.067	0.009
Indirect bilirubin, μmol/L	3.55 (2.65-6.80)	4.20 (3.70-5.50)	4.20 (3.23-5.58)	0.809	0.877	0.450
Triglyceride, mmol/L	1.52 (1.01-2.17)	1.01 (0.86-2.12)	1.55 (1.03-2.19)	0.843	0.336	0.261
Total cholesterol, mmol/L	4.45 (3.96-5.09)	4.38 (4.03-4.56)	4.69 (4.06-5.34)	0.779	0.376	0.372
High-density lipoprotein-cholesterol, mmol/L	1.16 (1.02-1.36)	0.94 (0.90-1.01)	1.17 (0.98-1.29)	0.758	0.038	0.001
Low-density lipoprotein-cholesterol, mmol/L	2.17 (1.84-2.71)	2.75 (2.54-2.86)	2.77 (1.90-2.98)	0.322	0.877	0.043
Lactate dehydrogenase, U/L	194.50 (153.75-225.75)	187.00 (169.00-204.00)	169.50 (156.00-179.75)	0.291	0.154	0.593
Hypersensitive C-reactive protein, mg/L	1.18 (0.46-2.86)	0.65 (0.29-2.36)	0.92 (0.64-1.57)	0.779	0.577	0.347
Modification of diet in renal disease equation, ml/min	-	101.90 (93.90-109.80)	99.10 (96.80-109.00)	-	0.849	-

RS: Recovered from symptomatic infection, RA: Recovered from asymptomatic infection, HC: Healthy controls.

Supplemental Table S3. Peptide constitution of peptide library used for T cell ELISpot.

Peptides library	Sequence		Number of peptides
SARS-CoV-2 S1 scanning pool	(15mers with 11 aa overlap)		166 peptides
SARS-CoV-2 S2 N defined peptide pool	1	LIDLQELGKY	41 peptides
	2	GTTLPKGFY	
	3	VTPSGTWLTY	
	4	ILLNKHID	
	5	GMSRIGMEV	
	6	ALNTPKDHI	
	7	LALLLLDRL	
	8	LLLDRLNQL	
	9	LQLPQGTTL	
	10	ALNTLVKQL	
	11	LITGRLQSL	
	12	NLNESLIDL	
	13	RLNEVAKNL	
	14	VLNDILSRL	
	15	VVFLHVTYV	
	16	GSFCTQLNR	
	17	GVVFLHVTY	
	18	AQALNTLVK	
	19	ASANLAATK	
	20	SLIDLQELGK	
	21	SVLNDILSR	
	22	VQIDRLITGR	
	23	KTFPPTEPKK	
	24	SASAFFGMSR	
	25	ATEGALNTPK	
	26	LSPRWYFYY	
	27	APHGVVFLHV	
	28	APSASAFFG	
	29	LQIPFAMQM	
	30	GRLQSLQTY	
	31	MEVTPSGTWL	
	32	MEVTPSGT	
	33	AQFAPSASAFFGMSR	
	34	YKTFPPTEPKKDKKKK	
	35	MAYRFNGIGVTQNVLY	
	36	QLIRAAEIRASANLAATK	

	37 QALNTLVKQLSSNFGAI<br>38 IDRLITGRLQSLQTY<br>39 IDAYKTFPPTEPKKD<br>40 MSRIGMEVTPSGTWL<br>41 VLQLPQGTTLPKGFY	
SARS-CoV-2 S N M O defined peptide pool	1 CTFEYVSQPFLMDLE<br>2 EFVFKNIDGYFKIYS<br>3 KHTPINLVRDLPQGF<br>4 NLVRDLPQGFSALEP<br>5 YAWNRKRISNCVADY<br>6 GVSPTKLNDLCFTNV<br>7 GGNYNYLYRLFRKSN<br>8 YLYRLFRKSNLKPFE<br>9 VVLSFELLHAPATVC<br>10 GPKKSTNLVKNKCVN<br>11 SVTTEILPVSMTKTS<br>12 STECSNLLLQYGSFC<br>13 NLLLQYGSFCTQLNR<br>14 NFSQILPDPSKPSKR<br>15 TDEMIAQYTSALLAG<br>16 GINASVVNIQKEIDR<br>17 LIDLQELGKYEQYI<br>18 YEQYIKWPWYIWLGF<br>19 MSDNGPQNQRNAPRITF<br>20 NQRNAPRITFGGPSDSTG<br>21 DQIGYYRRATRRIR<br>22 MKDLSPRWYFYYL<br>23 LSPRWYFYYLGTGPEAGL<br>24 AFFGMSRIGMEVTPSGTW<br>25 GMEVTPSGTWLTYTGAIK<br>26 TWLTYTGAIKLDDKDPNF<br>27 PNFKDQVILLNKHIDAYK<br>28 LLNKHIDAYKTFPPTEPK<br>29 LLESELVIGAVILRGHLR<br>30 GAVILRGHLRIAGHHLGR<br>31 LRIAGHHLGRCDIKDLPK<br>32 PKEITVATSRTLSYYKL<br>33 TSRTLSYYKLGASQRVA<br>34 IGNYKLNTDHSSSSDNIA<br>35 YFLCWHTNCYDYCIPY<br>36 KDCVVLHSYFTSDYYQLY<br>37 YFTSDYYQLYSTQLSTDTGV<br>38 GVEHVTFFIYNKIVDEPEEH<br>39 LITLATCELYHYQECVR	47 peptides

	40 FHPLADNKFALTCFSTQF<br>41 DGVKHVYQLRARSVSPKL<br>42 ILLNKHID<br>43 MEVTPSGTWL<br>44 QLIRAAEIRASANLAATK<br>45 SPRWYFYYL<br>46 YLGTGPEAGL<br>47 YYLGTGPEA	

Supplemental Table S4. Key reagents.

Reagent or resource	Source	Identifier
**Chemicals, Peptides, Recombinant Proteins and Culture medium**		
Fixable Viability Dye eFluor 780	eBioscience	65-0865-14
PHA-L Solution (500*)	eBioscience	00-4977-93
1M HEPES	Solarbio	H1095
PBS	Hyclone	SH3025601
Streptavidin-APC	Biolegend	405207 / 100 μg
GolgiStop	BD Biosciences	554724
GolgiPlug	BD Biosciences	555029
SARS-CoV-2(2019-nCoV) Spike S1-His Recombinant Protein, Biotinylated	SinoBiological	40591-V08H-B-20μg
DMSO	Sigma	D2650-5*10 ml
SARS-CoV-2 Spike peptide library*	GenScript	RP30020
SARS-CoV-2 Nucleocapsid peptide library*	GenScript	RP30013
SARS-CoV-2 Membrane peptide library*	GenScript	RP30022
Sodium Pyruvate 100 mM Solution	Gibco	11360070
2-Mercaptoethanol	Gibco	21985023
BASIC RPMI 1640 Medium	Gibco	C11875500BT
MEM NEAA,100×*	Gibco	11140050
Penicillin-Streptomycin,Liquid	Gibco	15140122
Ficoll-Hypaque gradient	GE Healthcare Life	17144003
**Critical Commercial Assay Kits**		
fixation/permeabilization solution kit	BD Biosciences	554714
SARS-CoV-2(2019-nCoV) Spike S1 Antibody Titer Assay Kit	SinoBiological	KIT003
IFN-γ Matched ELISA Antibody Pair Set, Human	SinoBiological	SEKA11725-5Plates
ELISA Supplemental Solution Set	SinoBiological	SEKCR02-5Plates
Human IFN-γ SARS-CoV-2 ELISpotPLUS kit (HRP), strips	Mabtech	3420-4HST-P1-1
Human IgG SARS-CoV-2 RBD ELISpotPLUS(HRP)	Mabtech	3850-4HPW-R1-1
**Antibody**	**Source**	**Clone Number**
**Human**		
Anti-CD28	eBioscience	16-0289-85-500ug
PerCP/Cyanine5.5 anti-human	Biolegend	HIT3a

CD3		
PE/Cyanine7 anti-human CD4	Biolegend	A161A1
FITC anti-human CD8a	Biolegend	RPA-T8
Pacific Blue™ anti-human CD45RA	Biolegend	HI100
PE anti-human CD197 (CCR7)	Biolegend	G043H7
Brilliant Violet 510™ anti-human HLA-DR	Biolegend	L243
PE anti-human CD38	Biolegend	HB-7
APC anti-human CD134 (OX40)	Biolegend	Ber-ACT35 (ACT35)
FITC anti-human CD154(CD40L)	Biolegend	24-31
Pacific Blue™ anti-human CD69	Biolegend	FN50
PE/Cyanine7 anti-human CD137 (4-1BB)	Biolegend	4B4-1
FITC anti-human CD185 (CXCR5)	Biolegend	J252D4
Brilliant Violet 510™ anti-human CD183 (CXCR3)	Biolegend	G025H7
PE anti-human CD196 (CCR6)	Biolegend	G034E3
Brilliant Violet 510™ anti-human IFN-γ	Biolegend	B27
PerCP anti-human CD19	Biolegend	4G7
FITC anti-human CD21	Biolegend	Bu32
Pacific Blue™ anti-human CD27	Biolegend	M-T271
Brilliant Violet 510™ anti-human CD138 (Syndecan-1)	Biolegend	MI15
PE/Cyanine7 anti-human IgG Fc	Biolegend	HP6017

Peptide library preparation: Dissolve the lyophilized peptide pool by addition of 40 µl DMSO to the vial. Then add 85 µl PBS, mix and aliquote and store at -20℃ or below. This stock solution will have a concentration of 200 µg/ml of each peptide.
